# Efficacy and Safety of Fevipiprant in Asthma: A Review and Meta-Analysis

**DOI:** 10.7759/cureus.24641

**Published:** 2022-05-01

**Authors:** Abdullah Jahangir, Saud Bin Abdul Sattar, Muhammad Rafay Khan Niazi, Marwah Muhammad, Ahmad Jahangir, Syeda Sahra, Muhammad Ans Sharif, Muhammad Yasir Anwar, Michel Chalhoub

**Affiliations:** 1 Medicine, Staten Island University Hospital, Staten Island, USA; 2 Internal Medicine, Staten Island University Hospital, New York, USA; 3 Internal Medicine, Staten Island University Hospital, Staten Island, USA; 4 Internal Medicine, Jinnah Hospital, Lahore, PAK; 5 Internal Medicine, Mayo Hospital, Lahore, PAK; 6 Medicine, Mayo Hospital, Lahore, PAK; 7 Internal Medicine, BronxCare Health System, Lahore, PAK; 8 Critical Care, Staten Island University Hospital, Staten Island, USA

**Keywords:** s: asthma, fevipiprant, asthma exacerbation, prostaglandin d2 (pgd2), prostaglandin d2 receptor 2 (dp2)

## Abstract

Fevipiprant is a non-steroidal oral prostaglandin D2 (PGD2) receptor 2 antagonist that reduces bronchial wall inflammation, possibly improving clinical outcomes in the asthmatic population.

A systemic review search was conducted on PubMed, Embase, and Central Cochrane Registry. Randomized clinical trials were included with Fevipiprant as an intervention arm compared to placebo. For continuous variables, the standardized mean difference, and for discrete variables, Mantel-Haenszel Risk Ratio (MH Risk ratio) was used for analysis. Confidence interval of 95% and p-value < 0.05 was considered significant. The analysis was done using a random-effects model irrespective of heterogeneity. Heterogeneity was evaluated using the I2 statistic.

A total of five articles, including seven trials, were included in the analysis. There was significant increase in post-bronchodilator forced expiratory volume in one second (FEV1) 0.249 (0.157-0.341), p<0.001 and pre-bronchodilator FEV1 0.115 (0.043 to 0.188), p=0.002. A decrease in asthma control questionnaire (ACQ) score of -0.124 (-0.187 to -0.062), p<0.001, was reported. Statistically significant asthma exacerbation reduction was reported in the high eosinophil count population with a daily dose of 450mg 0.77 relative risks (RR) (0.61-0.97). There was a positive deviation toward Fevipiprant 450mg dose for asthma reduction in the overall population, but it was not statistically significant. Fevipiprant produced a slight statistically significant reduction in asthma exacerbations in the high eosinophil count population with favorable deviation in the overall population. It significantly increased pre-and post-bronchodilator FEV1 and improved ACQ scores in treated patients. The benefits, though statistically significant, failed to translate into clinical importance.

## Introduction and background

Asthma is a heterogeneous inflammatory disorder of the airways, usually described as intermittent respiratory symptoms (wheeze, shortness of breath, chest tightness, and cough) associated with variable expiratory airflow limitation. Pathogenesis includes airway narrowing due to edema, sub-epithelial fibrosis, smooth muscle hypertrophy/constriction, and mucus hyper-secretions in response to triggers.

The worldwide prevalence of asthma is around 235 million [1,2]. The Global Burden of Disease (GBD) report of 2018 estimates that asthma accounts for approximately 420,000 deaths per year worldwide [3]. The death rate of asthma in the United States increased from 1982 to 2001 and later decreased, consistent with the international data [4]. For example, the overall mortality rate was 15.09 per million in 2001 and 9.86 per million in 2017 [5]. The estimated healthcare-associated yearly cost of asthma is $50.1 billion, with the hospital stays being the most significant contributor [6].

Severe forms of asthma, step 4 and step 5 of the GINA classification (Global Initiative for Asthma) are believed to be resistant phenotypes of asthma syndrome that respond poorly to regular asthma medications, especially glucocorticoids [7,8]. Given the complex underlying bronchial pathological inflammation pathways in asthmatic patients, asthma appears to be a clinical syndrome with multiple phenotypes and underlying mechanisms [9]. One underlying inflammatory pathway involves prostaglandin D2 receptor 2 (DP2) on multiple inflammations mediated cells like mast cells, eosinophil cells, and T helper type 2 cells. DP2 receptors are also called chemoattractant receptor-homologous molecules (CRTH2). This novel pathway is now the focus of multiple new oral drugs.

Oral therapies might have a superior hand to inhaled therapies as they are more prone to poor adherence and improper techniques [10,11]. This review will discuss and analyze one of the novel oral agents, "Fevipiprant," a non-steroidal once-daily oral tablet that blocks the DP2 receptor pathway [12]. The receptor plays a vital role in inducing and amplifying the inflammatory cascade, ultimately leading to structural airway damage. In our review, we analyzed the trials to understand better the efficacy and safety of Fevipiprant in the asthmatic population.

## Review

Materials and methods

**Figure 1:**
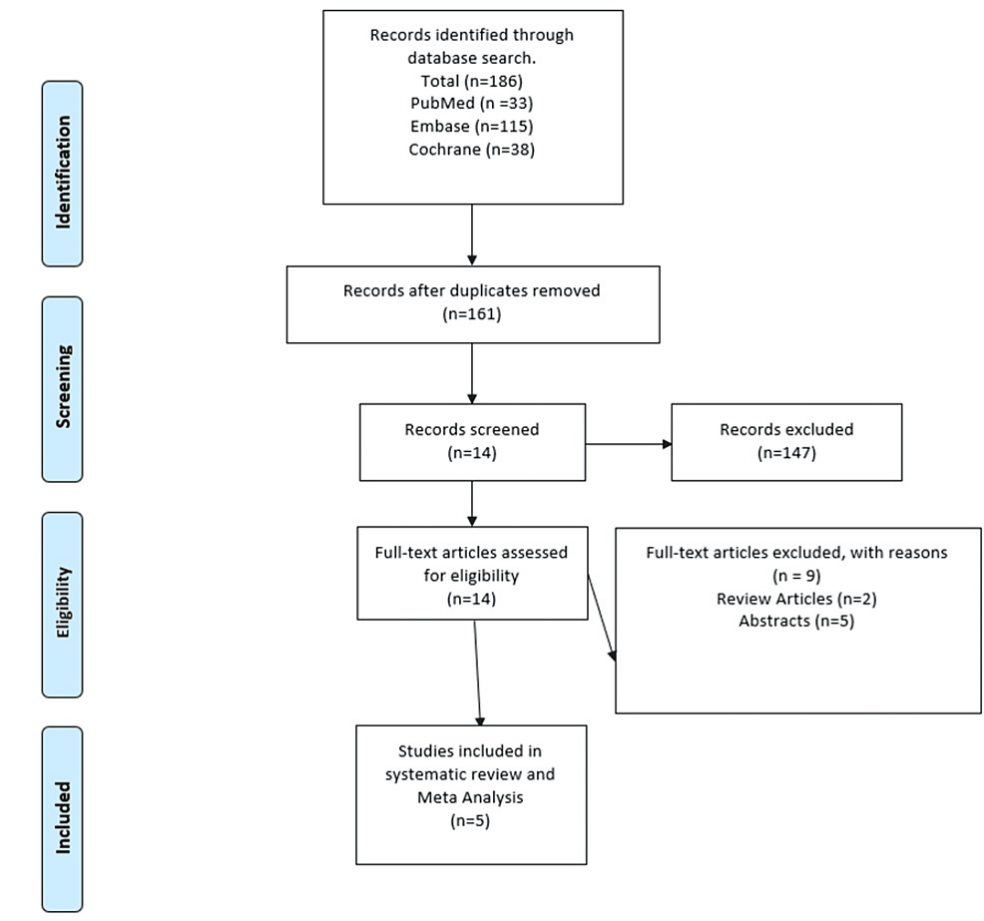
PRISMA figure for studies selected in meta-analysis.

The databases accessed were Cochrane Central Registry of Clinical Trials, Embase, and PubMed. Search terms used were Asthma, Fevipiprant, and QAW039. The deadline for publication was set as June 1, 2021. The inclusion criteria included papers in which: were randomized control trials on Fevipiprant against placebo in asthma patients; enrolled patients with an age greater than 12 years; were available in the English language without restrictions on the date or status of publications. Those papers which did not meet the above criteria were excluded. The Prisma guidelines have been illustrated in Figure 1.

Trial Selection and Evaluation

Two authors independently reviewed all articles and abstracts and excluded irrelevant articles. The risk of bias for selected papers was assessed using Cochrane collaborative tool and classified as high, uncertain, and low.

Data Extraction:

Information was extracted using a pre-specified extraction table. Data was extracted from trials reading through text and tables, and a second author reviewed the data collected to ensure the accuracy of the information. The extracted data included the number of patients, change in FEV1, change in Asthma control questionnaire (ACQ) scores, asthma exacerbation, and side effects, including headaches, viral URTI (upper respiratory tract infections), serious adverse events/SAE (defined as a congenital anomaly, significant or persistent disability, prolonged illness or life-threatening/ fatal circumstances), and nasopharyngitis.

Statistical Analysis

The meta-analysis was performed using the Comprehensive Meta-analysis software version 3. We calculated the standardized mean difference in continuous variables for treatment effect measurement, while the MH risk ratio was calculated for discrete variables. Standard errors were calculated using a 95% confidence interval, and a p-value of 0.05 was used for statistical significance. A random model was used irrespective of heterogeneity for consistency. Heterogeneity was evaluated using the I2 statistic; heterogeneity less than 40 was considered low, 40-60 as moderate, and above 60 as high.

Literature Search

A total of 186 articles were identified in the initial search. After the removal of duplicates, we filtered 161 articles. The first screening excluded 147 articles. We analyzed the full texts of 14 studies. Of the removed articles, five were abstracts, two were reviews, one did not report any clinical data, and one article contained only supplemental data from a trial. Five studies were included in the final analysis. Two studies reported results from two trials each, so we included seven trials with a total of 4784 patients in the review and meta-analysis. The main characteristics are given in Table 1 [13] (GB001 is another potent DP2 pathway antagonist under investigation right now for the control of asthma [14] and is not included in this meta-analysis) [15-18].

**Table 1:** Characteristics of the studies included.

Study	Study type	Participants	Inclusion	Exclusion	Primary Outcome	Secondary Outcomes	
Brightling 2020 [13]	Two-Phase 3 double-blind multicenter RCTs LUSTER 1 and LUSTER 2	LUSTER 1 Fevipiprant 150mg (n=301) vs Fevipiprant 450 (n=295) vs Placebo (n=298)	Age>12 Uncontrolled Asthma on dual or triple therapy with medium or high dose inhaled steroids Diagnosis of Asthma>24 months History of >=2 exacerbations in 12 months	Smoking within 6 months Greater than 10 pack-year smoking history Serious comorbidities History of malignancy	Number of moderate to severe asthma exacerbations in 52 weeks	Change in FEV1 at 52 weeks Change in ACQ-5 Change in AQLQ +12	
M. Castro 2021 [15]	Two-Phase 3 double-blind multicenter RCTs ZEAL 1 and ZEAL 2	ZEAL 1 Fevipiprant 150mg (n=339) vs placebo (n=336)	Age>12 Stable doses of medium- or high-dose ICS, low-dose ICS plus either LABA or LTRA, or medium-dose ICS plus LABA Diagnosis of Asthma>6 months History of >=2 exacerbations in 12 months	Clinically significant laboratory abnormality Other conditions leading to elevated eosinophils Serious comorbidities History of malignancy Pregnancy or Lactation	Pre-dose FEV1 at week 12	Change in AQLQ +12 Change in ACQ-5 Evening and morning PEF Nighttime asthma symptoms score Asthma exacerbation	
		ZEAL 2 Fevipiprant 150mg (n=352) vs placebo (n=350)					
		LUSTER 2 Fevipiprant 150mg (n=296) vs Fevipiprant 450 (n=294) vs Placebo (n=287)					
							Erpenbeck 2016 [16]	Double Blind Multicenter RCT	Fevipiprant 500 (n= 74) vs Placebo (n=84)	Age 18 to 65 Mild to moderate Allergic Asthma FEV reversibility (>12% or 200ml) and FVC >= 60% and <=85 at screening ACQ >= 1.5 at baseline	Smoking within 6 months Greater than 10 pack-year smoking history Women of childbearing age	Trough FEV1 at 4 weeks	Safety Peak FEV1 AUC 0-24h FEV1 curve ACQ-7 scores Changes in PEFR	
		Gonem 2016 [17]					Single Center Randomized Double-Blind RCT	Fevipiprant 450 (n= 30) vs Placebo (n=31)	Age >18 Diagnosis of Asthma as per GINA Sputum eosinophil count of >= 2% Current treatment with inhaled steroids ACQ >= 1.5 at baseline or one severe exacerbation in 12 months	Smoking within 6 months Greater than 10 pack-year smoking history Pregnancy or Lactation Serious Coexisting illness	change in sputum eosinophil levels at week 12	Change in ACQ-7 Change in FEV1 Change in AQLQS	
Bateman 2017 [18]	Single Center Randomized Double-Blind RCT	Multiple Dose Fevipiprant (n= 782) vs Placebo (n=136) vs Montelukast (n=139)	Age 18-65 on ICS therapy Reversible airway obstruction or AHR showed by a test in last 5 years FEV1 40-80% of predicted Allergic status by history, skin test or positive IgE ACQ >=1.5	Smoking Life-threatening asthma including hypercapnia, prior intubation, respiratory arrest, or seizures from asthma Prolonged QTc>450	Trough FEV1 at 12 weeks	Change in ACQ and JACD Onset of Efficacy by Spirometry and ACQ Dose-response relationship with FEV1 Compare the efficacy of Montelaukast and Fevipiprant to placebo	

Results

Risk of Bias

**Figure 2:**
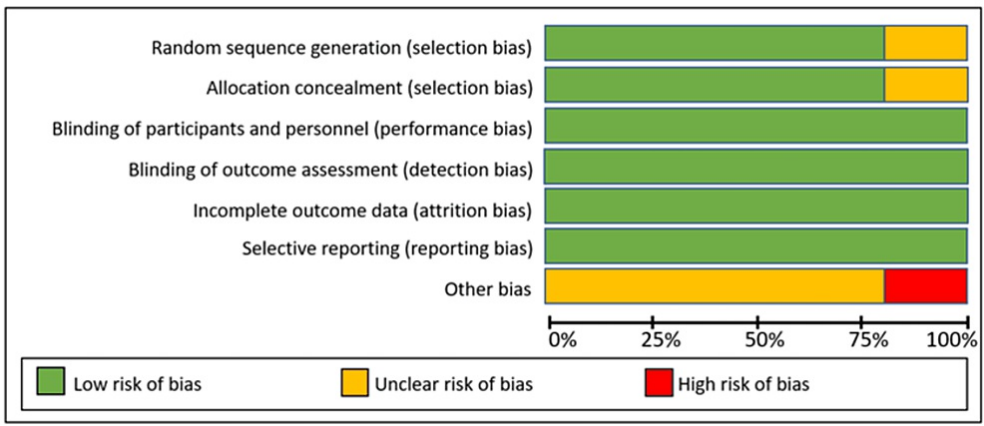
Risk of bias in studies included.

**Figure 3:**
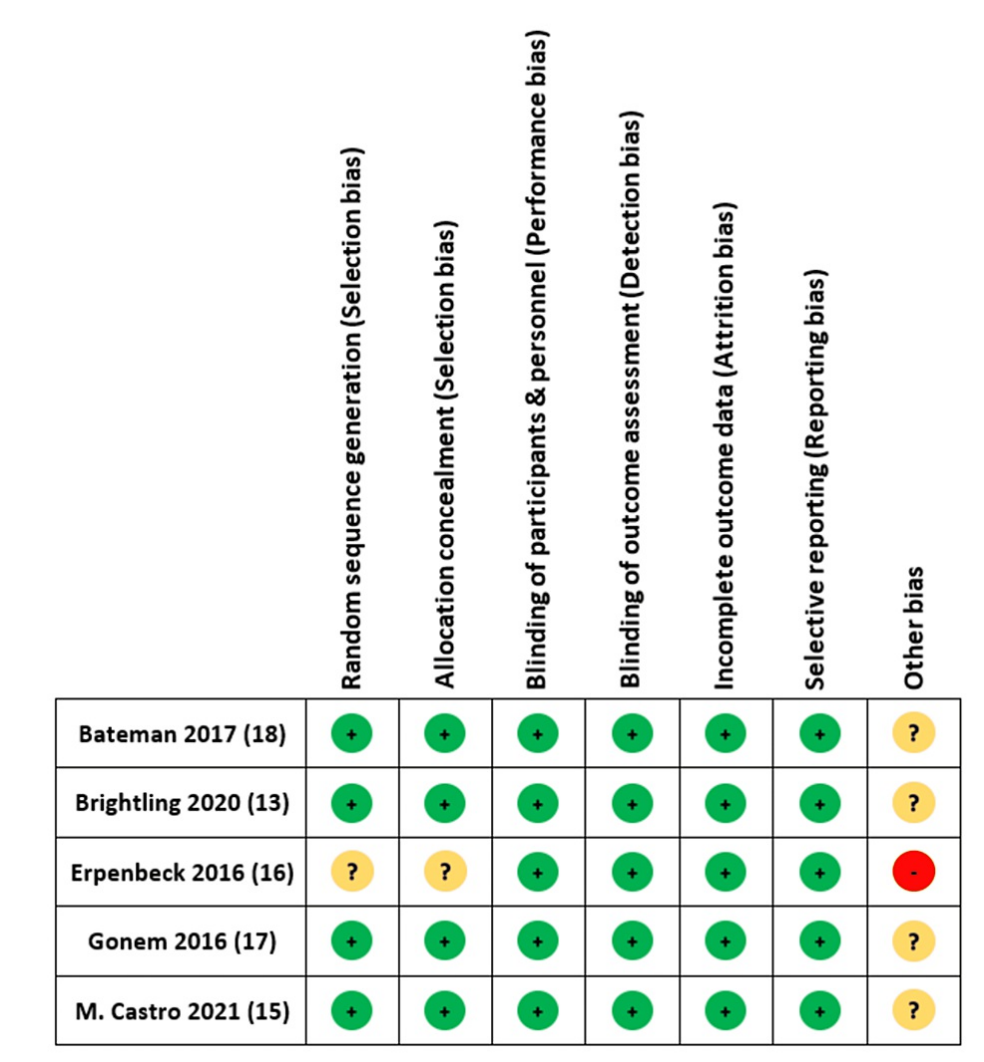
Assessment of risk of bias in studies included. References: [13,15-18]

The results of the risk of bias are shown in Figures 2, 3.

Results of Quantitative Analysis

**Figure 4:**
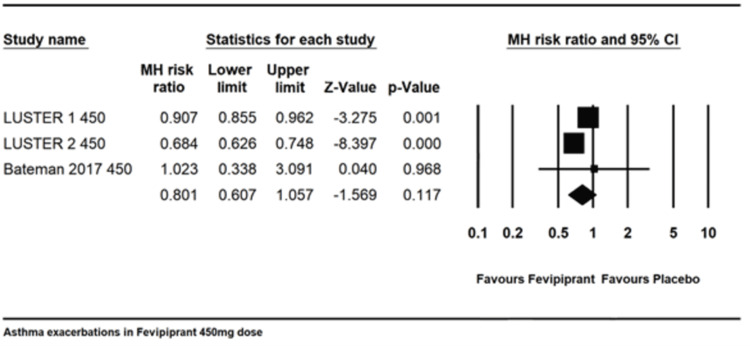
Asthma exacerbation for 450 mg dosing. References:  [13,18]

Asthma exacerbations: The overall exacerbations in 450mg daily dose. Three trials [13,18] reported an asthma exacerbation rate with 450mg dose with a combined MH risk ratio of 0.801 (0.607-1.057) with an I2 of 93.37. There was a substantial deviation towards Fevipiprant but it was not statistically significant (Figure 4).

**Figure 5:**
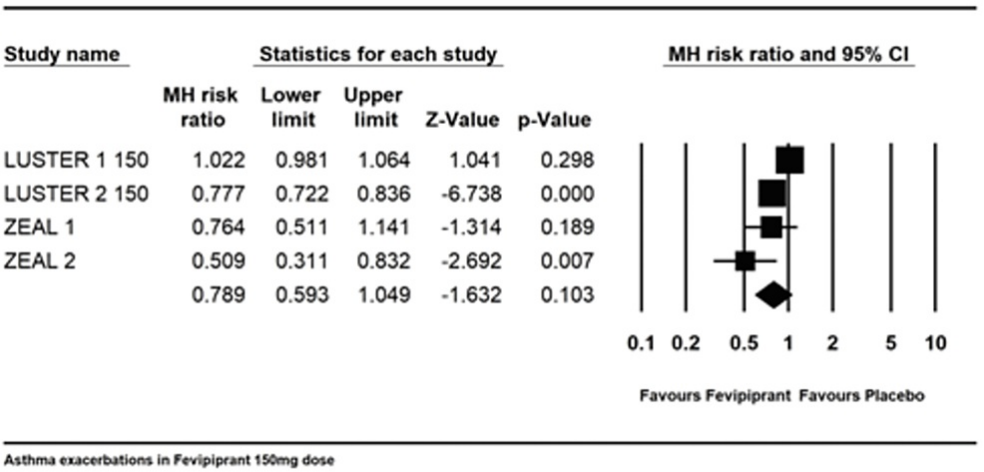
Asthma exacerbation in 150 mg dosing References: [13,15]

Overall exacerbations in 150mg daily dose: Two studies [13,15] with four trials reported asthma exacerbation rate with 150mg dose with a combined MH risk ratio of 0.789 (0.593-1.049) with an I2 of 96.18. As with the 450mg dose, there was a substantial deviation towards Fevipiprant but was not statistically significant (Figure 5).

Asthma exacerbation in high eosinophil population: LUSTER 1 and LUSTER 2 studies [13] reported annualized asthma exacerbation rates in patients with elevated eosinophil count. In LUSTER 1, the relative risk of annualized asthma exacerbation for 150mg dose compared to placebo was statistically insignificant with RR 1.04 (0.77-1.41), p=0.80. Similarly, it was also insignificant for 450mg dose with RR 0.83 (0.61-1.14), p=0.51. In LUSTER 2, the RR for 150mg dose compared with placebo was statistically significant 0.69 (0.50-0.96), p=0.06, but at 450mg dose, RR 0.72 (0.52-1.01), p=0.11 was statistically insignificant.

When both trials were analyzed together, it reported a statistically significant combined RR of 0.77 (0.61-0.97) for 450mg daily dose compared with placebo, but in 150mg dose, RR was 0.86 (0.69-1.08).

**Figure 6:**
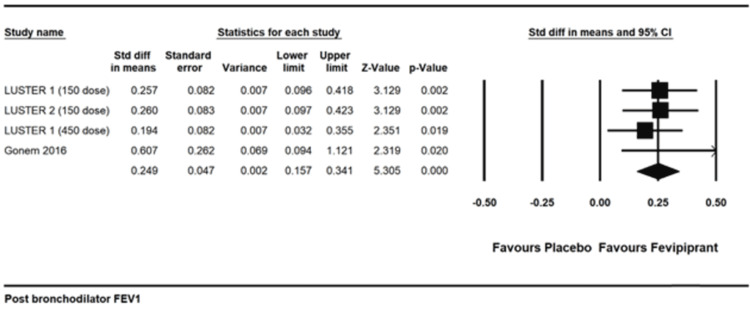
Post bronchodilator FEV1 References: [13,17]

Post-bronchodilator change in FEV1: There was a statistically significant increase in post-bronchodilator FEV1, standardized mean difference 0.249 (0.157 to 0.341), I2=0, p<0.001 [13,17]. The absolute mean difference was 77.261ml (49.113 to 105.409), p<0.001 (Figure 6).

**Figure 7:**
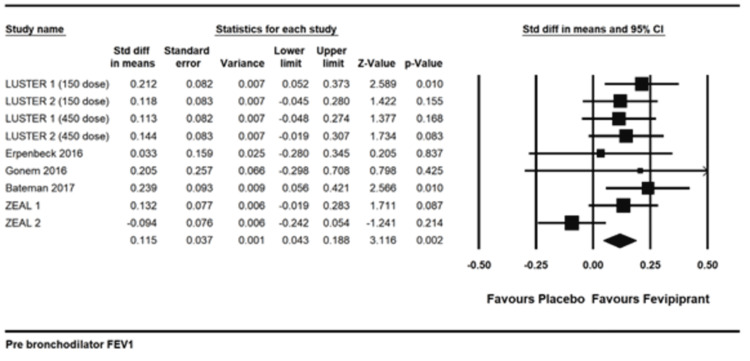
Pre bronchodilator FEV1 References: [13,15-18]

Pre-bronchodilator change in FEV1: There was a statistically significant improvement in pre-bronchodilator FEV1, standardized mean difference 0.115 (0.043 to 0.188) I2= 29.64, p=0.002 [13,15-18]. The absolute mean difference was 41.301ml (12.586 to 70.016), p=0.005 (Figure 7).

**Figure 8:**
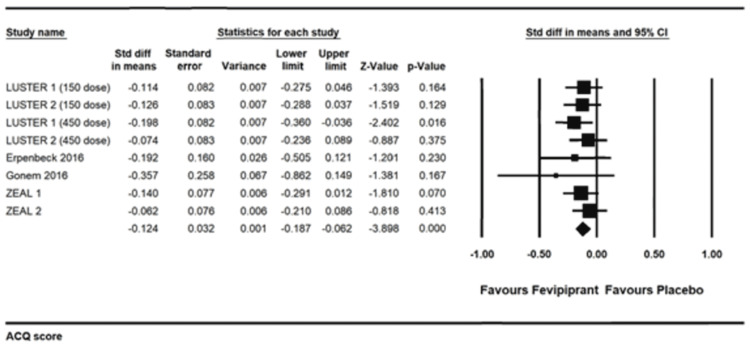
Asthma control questionnaire (ACQ) score. References: [13,15-18]

ACQ score: There was a statistically significant improvement in ACQ score, standardized mean difference -0.124 (-0.187 to -0.062) I2=0, p<0.001 [13,15-18]. The absolute mean difference was -0.109 points (-0.165 to -0.054), p<0.001 (Figure 8).

**Figure 9:**
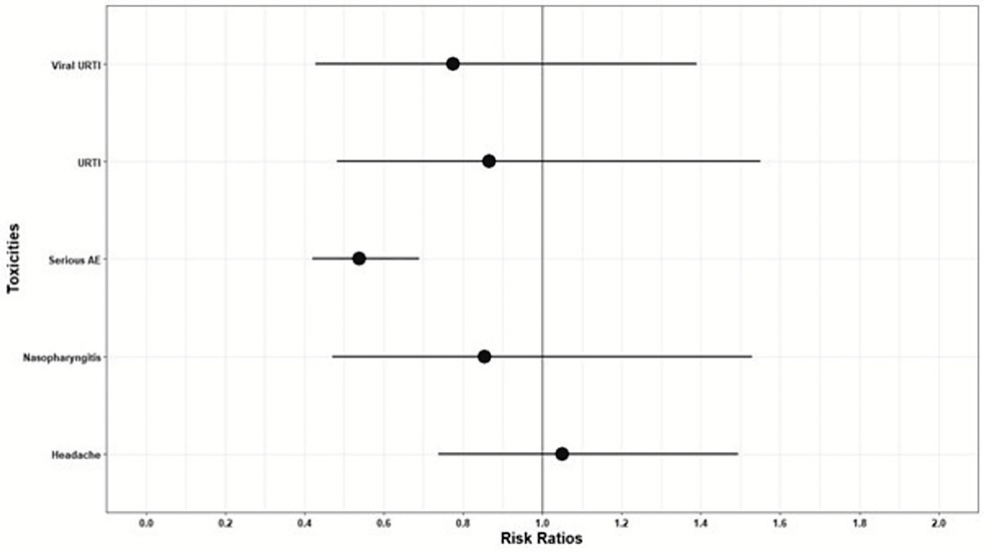
Risk Ratios (viral upper respiratory tract infections, upper respiratory tract infection, serious adverse effects, nasopharyngitis, headache)

Side effect profile: The side effect profile is illustrated in figure 9.

Summary of Results: Fevipiprant was associated with a statistically significant increase in pre-and post-bronchodilator FEV1 and significantly improved ACQ scores. It was associated with a statistically significant decrease in asthma exacerbation in the high eosinophil count population when the combined analysis was done at a 450mg dose. However, individual trials failed to show any clinical benefit. Even though there was a slight deviation toward reducing asthma exacerbation in the overall population, the results were not statistically significant.

Discussion

The DP2 antagonists have caused excitement in the medical community since their inception. High hopes are pinned on the class of drugs, given the ease of use and promise of significant efficacy. Novartis announced two phase-III trials on December 16, 2019, LUSTER-1 and LUSTER-2 of their novel drug Fevipiprant [13]. Results fell well short of expectations, and outcomes lacked clinical significance. Similarly, the recent results from ZEAL-1 and ZEAL-2 also failed to show any significant difference in multiple endpoints. The disappointment stemming from the trials has been palpable in the pharmaceutical industry. There is a question mark whether it is the failure of a single drug or the whole class of DP2 antagonists.

We performed this meta-analysis and systemic review by combining all trials on Fevipiprant to enhance the power of the findings. The study aims to understand whether the DP2 pathway promises future drug development, which can utilize the pathway better to attain better clinical results.

This review showed statistically significant improvement of pre-and post-bronchodilator FEV1 and modest improvement in ACQ scores. Even though the pooled data on the asthma exacerbation rate remained disappointing, there was a slight deviation favoring Fevipiprant that was not statistically and likely not clinically significant. The findings highlight that targeting the DP2 receptor pathway significantly affects lung function by improving pre and post bronchodilation FEV1, which may be a promising target for future studies. 

The major strength of our study is the significantly higher patient population analyzed and highlighting the significant physiologic improvements in lung function. Given that it is a retrospective study of heterogeneous studies, it is also subject to significant limitations. One of the biggest limitations is variable follow-up times leading to high heterogeneity and possible bias in results. It is also important to note a minimally significant change in ACQ scores even though ACQ scores were improved statistically significantly, the standardized mean difference of -0.124 (-0.187 to -0.062), p<0.001, in clinical settings, is at least 0.5. Thus, we can infer that the modest statistical significance translates poorly to improved quality of life and control of asthma measured by ACQ score.

**Figure 10:**
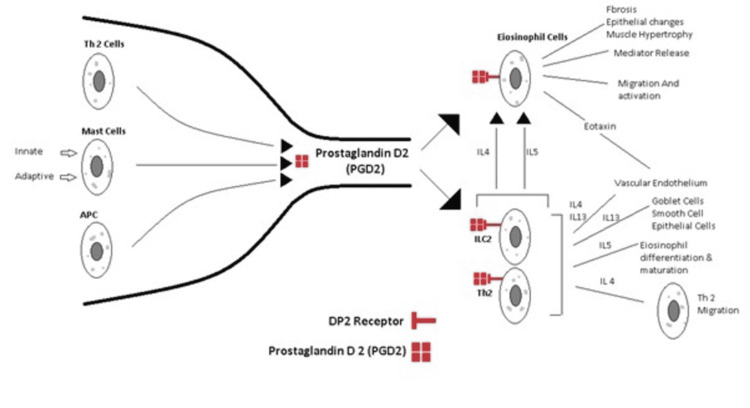
Inflammatory cascade highlighting the positioning of PGD2. Illustration created by the authors.

The pathology of asthma involves inflammatory cells like T helper type 2 (TH2), mast cells, and eosinophil cells which produce prostaglandin D2 (PGD2), a lipid mediator and a target of our primary drug discussed in this review. Prostaglandin D2 activates (CRTH2) receptors, leading to a fountain of inflammatory mediators that lead to chemotaxis and degranulation of inflammatory cells like basophils, ILC2, and eosinophils and TH2 cells. PGD2 is acting essentially as a bottleneck step in the inflammatory cascade. Theoretically, if we can stop this crucial step, we will stop further triggering the inflammatory cascade (Figure 10). GB001 is another potent DP2 pathway antagonist under investigation right now for the control of asthma [14].

## Conclusions

The benefits with the DP2 inhibitors appear to be modest at best, statistically, and likely clinically insignificant given current data. The findings did highlight that targeting the DP2 receptor pathway significantly affects lung function by improving pre and post bronchodilation FEV1, which may be a promising target for future studies. But despite some evidence of bronchodilation, it fails to translate into a significant improvement in asthma scores or overall exacerbations. On the current evidence, we do not recommend routine use of Fevipiprant in the treatment of asthma. The avenue for DP2 receptor antagonist remains promising and further trials with other agents are in progress.
